# Uses of Phage Display in Agriculture: A Review of Food-Related Protein-Protein Interactions Discovered by Biopanning over Diverse Baits

**DOI:** 10.1155/2013/653759

**Published:** 2013-04-28

**Authors:** Rekha Kushwaha, Christina M. Payne, A. Bruce Downie

**Affiliations:** ^1^Department of Horticulture, Agricultural Science Center North, University of Kentucky, Room 308J, Lexington, KY 40546, USA; ^2^Seed Biology Group, University of Kentucky, Lexington, KY 40546, USA; ^3^Department of Chemical and Materials Engineering, University of Kentucky, Room 159, F. Paul Anderson Tower, Lexington, KY 40546, USA; ^4^Center for Computational Sciences, University of Kentucky, Lexington, KY 40506, USA; ^5^Department of Horticulture, University of Kentucky, Room 401A, Plant Science Building, Lexington, KY 40546, USA

## Abstract

This review highlights discoveries made using phage display that impact the use of agricultural products. The contribution phage display made to our fundamental understanding of how various protective molecules serve to safeguard plants and seeds from herbivores and microbes is discussed. The utility of phage display for directed evolution of enzymes with enhanced capacities to degrade the complex polymers of the cell wall into molecules useful for biofuel production is surveyed. Food allergies are often directed against components of seeds; this review emphasizes how phage display has been employed to determine the seed component(s) contributing most to the allergenic reaction and how it has played a central role in novel approaches to mitigate patient response. Finally, an overview of the use of phage display in identifying the mature seed proteome protection and repair mechanisms is provided. The identification of specific classes of proteins preferentially bound by such protection and repair proteins leads to hypotheses concerning the importance of safeguarding the translational apparatus from damage during seed quiescence and environmental perturbations during germination. These examples, it is hoped, will spur the use of phage display in future plant science examining protein-ligand interactions.

## 1. Introduction

Since its development by Smith [1], phage display has proven to be a powerful tool for protein interaction studies in Immunology, cell biology, drug discovery, and pharmacology. Phage display is one of the preeminent means by which scientists identify proteins having affinity for other molecules and has a staggering throughput capacity for screening with libraries with titers approaching 10^9^ virions per microliter. Its utility lies principally in generating molecular probes against specific targets and for the identification, analysis, and manipulation of protein-ligand (including protein-protein) interactions. Modern phage display libraries permit the sought attribute (namely, protein with affinity for a ligand (bait)) to be directly coupled to the DNA sequence encoding the protein in a nondestructive manner. Random DNA libraries, or those formed from cDNA after randomly priming mRNA, provide a host of different amino acid contexts that can translate into a continuum of affinities for the bait. Recovery of overlapping clones of a particular protein permits examination of this region of the protein, directing the experimenter to the specific site capable of binding the ligand. With the protein-binding site effectively located, this information can be used to predict target attributes that serve as the foundation of ligand-protein affinity, guiding future protein engineering efforts.

This technique, due to its simplicity and efficacy, has been responsible for discoveries of synthetic antibodies and molecular interactions and utilized in directed evolution. The applications of phage display for discovery of protein-ligand interactions have become increasingly complex as its utility has been recognized in a diversity of fields, including the identification of targets of bioactive molecules. For example, Huperzine A is a plant-produced, bioactive compound with multiple neuroprotective effects [2, 3]. Magnetic biopanning approaches have been used to identify some of the target pathways influenced by Huperzine A's pharmacological effects which are responsible for alleviating a host of dysfunctions, potentially including Alzheimer's disease [4]. 

Despite the utility of phage display, the technique has received less attention from plant scientists, with the exception of sustained programs developing antibodies to a host of different cell wall components [5], a topic discussed in other literature [6] and thus not examined here. However, phage display has much to offer other fields of plant research. This review surveys the applications of phage display in the discovery of protein-protein interactions in various fields of plant science concerned with maximizing crop plants' seed production and the utilization of the nutrients stored in seeds, from protecting crops from harmful pests to alleviating human allergenic reactions to seed storage proteins. 

Our objective in highlighting this literature is to heighten the awareness of plant biologists to the utility of the technique for more than antibody production alone. If successful, phage display should figure more prominently in the research of those plant scientists examining molecular interactions in the future.

## 2. Applications of Phage Display in Agriculture: Seed Production

Why focus on seed production? On a fundamental level, it is necessary to understand seed attributes as human reliance on seeds is so pervasive. Seeds are our major food source (70% of our diet [7, 8]); they are fodder for our livestock, a method of bulk food transport, storage, germplasm preservation, and a vehicle for technology delivery. It is imprudent not to understand more about how a seed fulfills its function as a propagule, a process on which we depend so utterly, yet about which we still know so very little [9, 10]. In addition to constituting the majority of humanity's food, recent additional uses for the energy stored in seeds (biofuels [11]) have periodically led to higher seed and commodity prices worldwide [12, 13]. While governments attempt to mitigate the negative impact of increasing staple food prices on the poor [12], demand for seed as food and biofuel feedstock and the land on which to produce it continues to increase [14]. The growing global population is projected to increase cereal consumption for food alone by a billion metric tons in the next 30 years (FAO, 2002, http://www.fao.org/docrep/004/y3557e/y3557e00.htm); yet yield losses due to unpredictable biotic and abiotic stresses are projected to increase [15]. These grim facts have added urgency to the requirement to improve understanding of all facets of seed production. It is imperative that we do this if we are to feed ourselves [16]. 

### 2.1. Phage Display Utilized in the Defense of Plants against Herbivores and Microbes

#### 2.1.1. Identification and Production of Superior Protease Inhibitors

**Figure 1:**
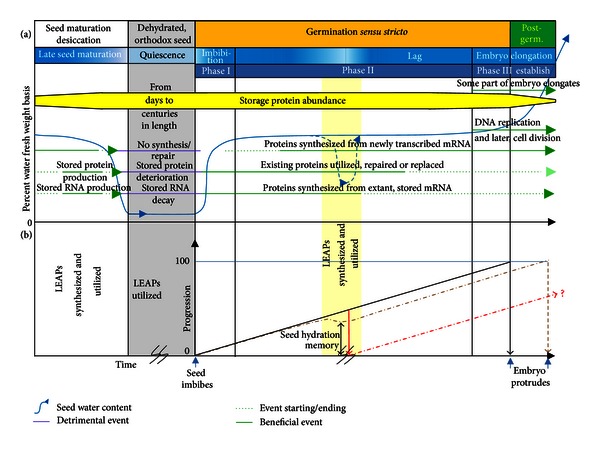
A graphic depiction of events occurring during the stages of late maturation, quiescence, and germination of orthodox seeds [27]. (a) Four stages during a plant's lifecycle commencing with seed maturation desiccation and ending with postgermination seedling establishment (Postgerm). Seed water content is represented by the solid blue line in the graph and is depicted as well by shades of blue in the background highlighting stages in the continuum encompassing late seed maturation, quiescence, and the three classical phases of water uptake during seed germination (imbibition, lag, and embryo elongation/seedling establishment (establish)). Phase III has been placed to span the completion of germination because turgor-driven embryo cell expansion, required to protrude from the seed, necessitates additional water uptake. The axis representing time has been broken during quiescence to emphasize that, although this period can last for centuries, certain species seeds remain viable [28, 29]. Events that are beneficial for the preparation of maturation desiccation or the resumption of growth are presented as green lines. Events occurring that are detrimental to the cellular constituents are depicted as purple lines. The commencement and termination of these events are signified by short-dashed lines. A drying event, followed by rehydration during germination, has been inserted as a long-dashed blue line. This region is also highlighted by yellow shading that depicts a period of high temperature stress. The abundance of the seed storage proteins is depicted as a yellow bar whose thickness is tapered at both ends to signify net accumulation during late embryogenesis and rapid hydrolysis during seedling establishment. (b) Late embryogenesis abundant protein (LEAP) synthesis and utilization during late seed maturation and quiescence. The overall progression of a non-dormant (quiescent) seed toward the completion of germination (100% progression) is depicted as a solid line commencing at the arrow (seed imbibes) on the time axis. To emphasize the capacity of the seed to preserve its physiology at a point above 0 progression (*y*-axis) during the dehydration/supraoptimal temperature event (dash-dotted brown line), the trajectory of progression deviates partially from that had no drying/thermal stress occurred. The red line, and the dash-dotted red progression trajectory emanating from it, portrays a seed without the capacity to preserve its physiology. The difference (double-headed arrow) is the seed hydration memory [30]. The only manifestation of the stressful event interrupting the progression of germination is a slightly delayed point on the time axis at which the embryo protrudes. A seed unable to maintain its physiology may or may not be capable of completing germination, hence the question mark. The production of the LEAPs and their utilization to presumably preserve the seed's physiology, post-imbibition, are indicated. The time axis is broken during the stressful event to signify its unknown duration. Graph adapted from Nonogaki et al. [10].

Protease inhibitors (PIs) are one defense system plants employ against herbivores and microorganisms [17]. PIs are a plant protection strategy that can attenuate nutrient assimilation in the insect gut or by microbes by inhibiting the activity of pest digestive proteases [18]. There are a large number of PIs used by plants as natural protection against pests [19]. PI production can be induced in the plant body by pest/pathogen attack through the jasmonic acid pathway [20], but are also subject to developmental regulation, their production being stimulated in storage tissues [21]. In seeds, PI transcription is stimulated by abscisic acid (ABA) (inhibitory to germination) and inhibited by gibberellic acid (GA) (stimulatory for germination) [22]. Thus, endogenous seed protease activity (responsible for storage protein breakdown for use by the establishing seedling) is reduced during the anabolic period of seed development, permitting unhampered accumulation of the storage proteins, while this hindrance is alleviated during the period of seedling establishment allowing access to energy and components constituting the storage proteins (Figure 1(a)). Reduction, through the NADPH-dependent thioredoxin h system, of specific disulfide bonds necessary to impart the PI with its inhibitory confirmation [23] also aids the removal of seed PI influence from establishing seedlings [24]. Typically, PIs are heat labile, permitting humans to acquire the full nutritional value of the seed storage proteins (some of which are protease inhibitors in their own right [25]) in cooked food that is denied to insects and microorganisms [26]. 

The plant usually encodes a considerable variety of PIs that are used to inhibit a wide range of pest proteases and isoforms within a protease class. Protease isoform prevalence in the insect can vary, exhibiting adaptability on the part of the pest in attempts to overcome this plant defensive mechanism [31–34]. Strategies using phage display to inform directed evolution [35, 36] or specific site-directed mutation [37] efforts to produce PIs with greater specificity [38] or affinity [39] for the pest protease active site aim at enhancing this natural means of protecting crops. The PIs are usually quite specific for their protease target [40], and phage display has been at the center of efforts to construct PIs with a greater range of targets. This enhanced generality includes biopanning for PI variants that can inhibit proteases of a diversity of insect pests [41]. Another facet of phage display-based protection enhancement takes the opposing strategy, endeavoring to identify PIs that are even more finely tuned to the target species (pest) protease class [42].

 These various attempts to use phage display to acquire novel PIs are geared toward providing a greater range of PIs affording protection to plants than is available to the conventional plant breeder. The development and identification of PIs with unique capabilities of downregulating the activity of specific pest proteases, through phage display or other means, will permit these plant protection mechanisms to augment those existing naturally in the plant. Stacking PIs with different protease target sites may help to broaden pest susceptibility while delaying the acquisition of resistance to the PIs [43, 44].

#### 2.1.2. Discovering Non-Protease Inhibitor Protective Peptides

 Phage display can identify peptides or proteins that have affinity for a vast array of molecules. Peptides with high affinity for proteins key to a pest's lifecycle can be disruptive to the pest's permanence or pathology [45]. Once identified, such peptides can be engineered and introduced into most crop plants for endogenous production, providing a novel line of defense against plant pests. Such specific, plant-contained, protective mechanisms may prove to be less damaging to off-target organisms in the crop environment than conventional pesticides [46]. Chemoreception-disruptive peptides selected from peptide libraries have been shown to decrease parasitism by nematodes, albeit at doses 3 orders of magnitude greater than the Aldicarb nematocide control [47]. Despite this much lower competence, the Aldicarb mimetic with high affinity to acetylcholinesterase, when produced *in planta*, was effective in reducing parasite load in potato by cyst nematodes [46] that are otherwise difficult to control due to their sessile habit and location, embedded in the plant roots. Therefore, *in situ* production of the mimetic with a lower efficacy counteracted this liability, resulting in nematode control, which is also the goal of the generally applied nematocide possessing greater potency but only a portion of which arrives at the site of action. Similarly, phage display identified peptides binding to zoospores of the fungal pathogen *Phytophthora capsici*. Many of the zoospore-binding peptides resulted in the premature encystment of the zoospore without any other inductive signal. In addition to aiding in the identification of zoospore-displayed receptors controlling encystment, the authors postulated that such peptides might represent a novel plant defensive mechanism [48]. Subsequently, decreased infection by this soil-borne fungus resulted when a protective peptide was expressed *in planta* in a form allowing its secretion into the rhizosphere [49].

#### 2.1.3. Uses in Plant Virology

Phage display has been used by various plant virologists in identification of peptides that bind to a pathogenic virus's coat protein. The phage display-isolated peptides were very specific and highly sensitive. At the very least, these have diagnostic potential as they can be produced as fusions with proteins that serve as an antigen for antibody-reporter molecule conjugates [50]. They may also constitute the basis for a novel, introduced disease resistance strategy. Peptides with high affinity and specificity for vital viral proteins could be identified, and subsequently, the capacity to synthesize these peptides may be introduced into plants. *In planta* peptide production might prevent viral proliferation in infected cells. Such a strategy has been used successfully with antibodies [51], but antibody folding usually requires an oxidizing environment conducive to forming specific intracellular disulfide bonds necessary for function [52]. Phage display-selected peptides may not be so exacting in their requirements [53]. Indeed, phage display-selected peptides capable of binding to a coat protein of the rice black streaked dwarf virus (RBSDV), when produced recombinantly for diagnostic purposes, have been shown to also disrupt proper coat protein folding and reduce the pathogenicity of RBSDV [54]. Phage display has also assisted in the elucidation of various host systems secunded to the virus to permit successful infection and replication. Using the viral replication enhancer protein, AC3 as bait, a phage library of random dodecapeptides fused to a coat protein was panned to identify interacting peptides that were then analyzed for homology to proteins from the model plant,* Arabidopsis thaliana*. The revelation of the pathways to which these proteins are integral has allowed a more sophisticated understanding of events required for successful viral lifecycle and the role of the multifunctional protein AC3 in events leading to virus-induced gene silencing [55].

#### 2.1.4. Identification of Immune Targets in Plants

Plants are known to have a very complex and diverse immune system against microbes [56]. The first active line of defense occurs at the plant cell surface when microorganism-associated molecular patterns (MAMPs) such as lipopolysaccharides, peptidoglycans, or bacterial flagellin are detected by pattern recognition receptors (PRRs). These PRRs are responsible for pattern-triggered immunity (PTI) in plants [57–59]. To circumvent PTI, adapted pathogens can deliver effector molecules directly into the plant cell. As a countermeasure, plants have developed corresponding resistance (R) proteins to recognize these effectors and their modified targets which results in effector-triggered immunity (ETI) [59]. Both PTI and ETI involve specific families of proteins but the distinction between both types is not yet clear. What is clear is that a large number of proteins participate in the immunity process. Rioja et al. used phage display to study these interactions and to identify *Arabidopsis* proteins able to bind bacterial pathogens [60]. For this, they constructed two phage-display libraries from the cDNA of microbe-challenged *Arabidopsis*. Recombinant phage displaying plant proteins capable of interacting with different species of *Pseudomonas* (the pathogen) were selected by biopanning using microbial cells as selection ligands. In this way, plant proteins involved in defense responses were identified and subsequently confirmed *in vitro* for the capacity to bind microbial cells. Using different strains of *Pseudomonas* as bait allowed discrimination between common bacterial receptors and specific targets of virulent or avirulent strains.

### 2.2. Applications in Cell Wall Research

 Interest in using cellulose and other plant cell wall components as feedstock for biofuel production continues to grow worldwide for a host of reasons. Current means of deconstructing cellulose polysaccharides to glucose for conversion to biofuels are less efficient and more expensive than practical for an industrially relevant process. One avenue being explored for more efficient conversion of cellulose to glucose is through enhanced enzymatic degradation. It has been demonstrated that some cellulases and hemicellulases retain their function when fused to a viral coat protein [61, 62]. These clones can subsequently be reengineered to alter (randomize) specific regions of interest imparting novel functionalities/affinities to the displayed enzyme combinatorially. The resultant library of phage displayed variant enzymes can then be screened over substrates/inhibitors to study the individual amino acids imparting the observed/desired property. 

Programs have also used phage display libraries to discover or improve upon carbohydrate binding modules focused on the use of these regions to enhance the binding affinity of the glycoside hydrolase/binding module construct to various crystalline morphologies, which may improve upon their productivity [63]. Additional uses include highly specific probes for cell wall constituents, which are critical to refining our understanding of plant cell wall construction [64–66].

Furthermore, a library of fungal endo-*β*-1,4-xylanase enzyme variants permitted the simultaneous assessment of the influence of many different individual residues on the affinity for xylanase inhibitor proteins [67]. Subsequent work has permitted the development of an endo-*β*-1,4-xylanase enzyme that retains its catalytic competence while being completely insensitive to xylanase inhibitor proteins found in wheat flour [68]. The fungal xylanase is used in the food industry to enhance nutritional value and properties, but its inactivation by the endogenous inhibitors found in the foodstuffs on which it is used has been a problem for the industry. Moreover, through a computational approach, the pH stability of the enzyme has now been greatly improved leading to an increase in its utility in the food preparation industry [69].

### 2.3. Phage Display Uses in Combating Allergies to Seed Storage Proteins

Almost 5% of humans have some form of food hypersensitivity [70]. Identified food allergens include the seed storage proteins that can induce a variety of allergic syndromes [71, 72]. Phage display has assisted in the rapid identification of antigens eliciting hypersensitive responses [73] including those previously uncataloged [74]. Once individuals suspect they are allergic to a particular food, a more sophisticated assessment of the component(s) in the food causing the allergic reaction is necessary if any alleviation is to be attained. Epitopes from a library of allergens from the food in question [75], panned over patient IgE, can rapidly and cheaply identify the specific allergen(s) causing the hypersensitive response [74]. For example, peanut allergies are quite common (~1% of the population of the USA [76]), are perceived to be increasing [77], and can be severe [78]. Phage display has been used to identify precisely what proteins are causing the hypersensitive reaction in peanut-sensitive patients [79], implicating the seed storage proteins as significant and accounting for 6 of the 8 allergens identified in peanut to date [80].

Similarly, “baker's asthma,” a common occupational affliction, was until recently only known to be caused by an allergic reaction to “flour” components. Phage display was used to identify a causal agent in wheat flour as native gliadin (33% of all cases) and, more specifically, *α*- and *β*-gliadin, which were causal in 12% of all Baker's asthma [81]. The use of such epitope display accurately identifies the causal agent of food allergies that, once identified, can be the subject of investigations aimed at rendering it less antigenic. Such an approach has been used in a program aimed at mitigating allergenic reactions in celiac disease.

Celiac (or also coeliac) disease affects approximately 1% of the human population [82]. It is induced by components in several cereal storage proteins in common use (bread, pasta, and beer). It is a complex disease with aspects of both autoimmune disease and food hypersensitivity [83]. In the autoimmune response, tissue transglutaminase (tTG) enzyme is targeted by self-antibodies but only after gluten ingestion when tTG is complexed with gluten [84, 85]. The enzyme deaminates the abundant glutamine residues, which can comprise up to ~35–40% of the amino acids constituting the *α*-gliadin component of gluten [86]. Antibodies are also specifically produced against tTG-deaminated gliadin fragments from gluten, a hallmark of food hypersensitivity [87]. 

Approaches to alleviate disease symptoms include attempts to block portions of gliadin using synthetic, high-affinity peptides, thus preventing tTG action/gliadin modification and subsequent formation of immunostimulatory epitopes. Phage display has played a critical role in the identification of the peptides possessing a strong affinity for gliadin. These act to first depress tTG activity against the gliadin substrate *in vitro* by steric hindrance, the eventual goal being to attenuate the autoimmune response by decreasing the association of the enzyme with its substrate, minimizing inflammation *in vivo* [88]. The second prong of this program is to cover the epitopes on gliadin, masking the protein fragments from the antibodies binding to them [89]. This program has passed the first several hurdles in the long road to providing a modicum of relief for celiac disease sufferers, including proof that the synthetic peptides act to block tTG activity against gliadin as did the phage-tethered peptides on which they were based, which does not necessarily follow [90]. The program awaits trials of the identified gliadin-binding peptides *in vivo*. In addition to their potential therapeutic uses, the various peptides, binding to different sites on the gliadin protein, [89] could provide valuable tools for researchers in the field of celiac disease.

### 2.4. Phage Display Identifies Protein Isoaspartyl Methyltransferase Substrates in the Stored Seed Proteome

The tTG-mediated alteration of gliadin glutamine residues, through deamidation, enhanced the antigenicity of gliadin fragments [91]. The proteins present in dry seeds are particularly susceptible to a host of nonenzymatic conversions, many of which are deleterious [92–99], and some of which may play a role in preparing the seed for the completion of germination upon rehydration [100]. Regardless, these conversions can also result in peptides that are recognized by the human immune system or are recalcitrant to hydrolysis. For example, spontaneous isoaspartyl formation is known to result in autoimmune responses [101] and interfere with peptide degradation [102] decreasing the nutritional value of ingested seed products [103] and, if sufficiently widespread in the stored proteome, would be disastrous for germination and seedling establishment [104, 105].

 Orthodox seeds [27] are capable of extreme dehydration allowing them to remain viable in extremes of temperature [106, 107] and in some instances, for centuries [28, 29]. This remarkable feat means that the seed proteome is at risk for deleterious alteration for the whole of this time as there is insufficient water present to effect repair. A prominent detrimental alteration is the conversion of L-Asn or L-Asp residues in proteins to succinimide that, upon water addition, usually converts to the unusual, uncoded amino acid, L-isoAsp [108–111]. In the imbibed state, isoAsp in proteins is recognized, methylated, and repaired by protein L-isoaspartyl methyltransferase (PIMT) [112, 113]. 

What proteins are most at risk for isoAsp formation or for which PIMT has highest affinity? Due to the labile nature of the labeled isoAsp and susceptibility of proteins to form isoAsp during rigorous extraction necessary to obtain samples, these identifications have not been facile [114–116]. Moreover, the abundance and susceptibility to damage of the seed storage proteins [93] have made identification of additional PIMT target proteins using extracts from seeds difficult [116]. 

**Figure 2:**
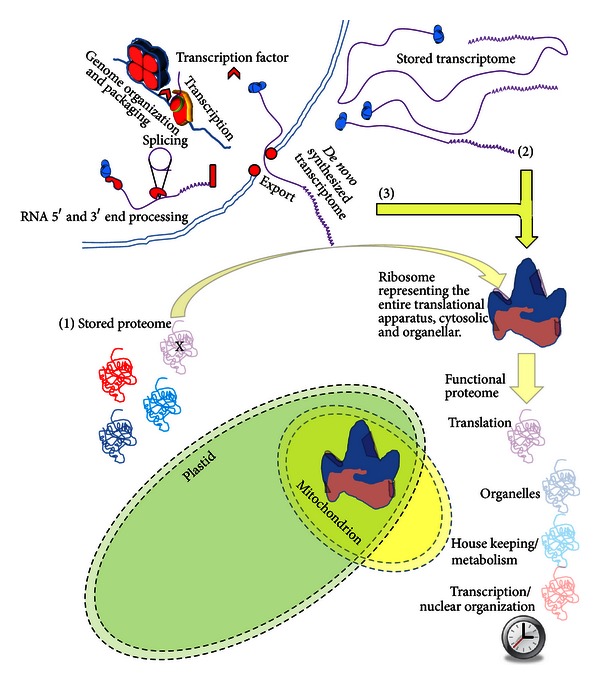
Those proteins essential to translation are the proteome's “Achilles' heel” for seed longevity. In the imbibed seed, there are three means by which functional proteins can be recruited into the newly reestablished, active metabolism. The proteins may be part of (1) the stored proteome that has survived maturation desiccation and subsequent rehydration with their function intact. New protein can be translated from either (2) the stored transcriptome consisting of mRNA, produced during seed maturation, that survived maturation desiccation/rehydration or (3) *de novo* transcribed mRNA. Only those proteins essential to translation *must* be present in the stored proteome, sufficiently numerous and in an active state following imbibition, to carry out translation (probably with an emphasis on self-replacement) if the embryo is to survive. Various classes of proteins are color coded according to their function (red: transcription/nuclear organization; light blue: House-keeping/metabolism; dark blue: organelles; purple: translation). The proteins essential to translation are depicted decorating the ribosome in the cytosol, or in those organelles with their own genomes. The dysfunction of the proteins essential for translation has been emphasized by their partial transparency and an “X” through the molecule representing this class in the stored proteome. A lack of translation results in the eventual demise of the entire proteome over time (partially transparent functional proteome).

An alternative approach used phage display to mitigate the influence of protein extraction on the generation of isoAsp while largely removing the seed storage proteins from the analysis [117, 118]. A group of proteins involved in aspects of translation were revealed as important substrates of PIMT in seeds. This led to the realization that the stored proteins essential for the translational apparatus must be especially important to protect from general dysfunction because there is no means of replacing them (or any other protein) from either the stored or *de novo* produced transcriptomes if translation is compromised in the majority of cells comprising a tissue and/or organelles [119, 120] present in cells (Figure 2).

### 2.5. Phage Display Identifies Late Embryogenesis Abundant Protein Client Proteins in the Seed

One of the targets recovered from the biopans over PIMT1 and not directly involved in translation was the seed maturation protein1 (SMP1; At3G12960), a Pfam (PF04927) SMP late embryogenesis abundant (LEA) protein homolog to the soybean (*Glycine max*) SMP, *Gm*PM28 (Glyma08G18400). LEA proteins [121, 122] are thought to assist anhydrobiosis (life without water), an attribute of many microorganisms, lichens, and some animals and plants [123–136]. This trait has underpinned agriculture for millennia [137, 138], allowing a portion of each seed harvest to be withheld, dehydrated, and hence, resistant to pathogen attack, and to establish the next crop, either the subsequent year or decades into the future [139].

The recovery of an LEA protein by PIMT1 was intriguing as it may indicate that this LEA protein needs protection from isoAsp formation by PIMT1 to retain its function, forming part of an interactive network of protein protective mechanisms extant in seeds. T-DNA insertional mutants of this LEA in two different *Arabidopsis* ecotypes were incapable of entering secondary dormancy when seeds were exposed to supraoptimal (40°C) germination temperatures for several days prior to being placed at permissive temperatures (25°C) [117]. Such a specific phenotypic manifestation of the loss of this LEA's function suggested it safeguarded a crucial subset of proteins involved in the proteomic memory of environmental conditions the seed has experienced thus far following imbibition (supraoptimal temperatures). High temperature and/or desiccation after a period of imbibition during which important environmental cues had been perceived and the transcriptome/proteome altered accordingly, but prior to radicle protrusion, would expose the proteome and the integrated environmental information it represents to deleterious conditions. This necessitates protective mechanisms be invoked to ensure the heat-stressed/dehydrated proteins retain their function so that germination can resume at the appropriate point at which it left off once the seeds are rehydrated [140]. Dubrovsky [30] referred to the capacity of seeds to resume germination from the point at which they had progressed prior to dehydration as the “seed hydration memory” (Figure 1(b)). 

The concept of the LEA proteins safeguarding environmental cues, acquired during the imbibed period and embodied in a heat-sensitive proteome, can be subsumed into their role of aiding the survival of water loss during maturation desiccation, quiescence or after imbibition [141, 142]. The dysfunction of some heat-labile molecule(s), when not protected by SMP1, results in a seed that cannot “remember” the supraoptimal temperature it has experienced and thus behaves inappropriately, completing germination immediately when removed to 25°C rather than entering thermal dormancy (Figure 1(b)).

It was necessary to ascertain with what target proteins the SMP1 LEA protein associates because these would be candidates for controlling the induction of secondary dormancy due to high heat [117] but this was not known. In fact, uncertainty exists regarding whether LEA proteins serve exclusively as general “spacer” molecules (“molecular shields” or crowders) that simply prevent deleterious aggregation upon water loss or if they can act as specific protectors of individual target molecules so-called “client molecules” [143–145]. Therefore, recombinant SMP1 and its soybean *Gm*PM28 homolog were used as bait in screens at two different temperatures and with two independently produced Arabidopsis seed, phage display libraries [146]. Biopanning over these recombinant LEA homologs demonstrated that the same protein clients, indeed the same region of the same protein clients, are consistently retrieved by both baits at two different temperatures [146]. The client proteins identified did not have a single target protein in common with the PIMT1 screens, yet those involved in translation were again prominent among the protected target proteins further entrenching the contention that protection of the proteins involved in translation is paramount for safeguarding the longevity of orthodox seeds (Figure 2).

## 3. Conclusions

Predictions of dire consequences for humanity if food (read seed) production is not drastically increased is a goad for researchers investigating seed production to endeavor to understand more of the complexities of this event. Frequently, the understanding sought lies at the level of protein-ligand or protein-protein interactions. In this regard, phage display has proved extremely useful for both the discovery of such interactions and their subsequent manipulation towards an end. This review has highlighted, for the first time, the impact phage display has had on agricultural research concerned with seed production. Efforts to safeguard the crop plant's capacity to produce seeds and to protect the seeds themselves for exclusive human use/consumption have successfully employed phage display. Phage display has aided in the production of enzymes specialized for use in food processing, making nutrients more readily available. It has also provided the means of specifically identifying the causal agent(s) of seed allergies, and indications are that it may be instrumental in providing the first means of mitigating the effects of a prominent seed-related ailment. The use of phage display has permitted insights into the seed's endogenous natural protective and repair mechanisms, allowing a more fundamental understanding of the events transpiring during late embryogenesis, quiescence, and germination; in short, what makes seeds so excellent in their role as propagules.
